# Supplementary material for: How many begomovirus copies are acquired and inoculated by its vector, whitefly (Bemisia tabaci) during feeding?
Source: PLoS One. 2021 Oct 26;16(10):e0258933. doi: 10.1371/journal.pone.0258933 (PMC8547624; doi:10.1371/journal.pone.0258933)
Supplement: S1 Table — (DOCX) [file pone.0258933.s001.docx]

**Supplementary Table 1** Copies of ToLCNDV and ChiLCV ingested and egested by individual *B. tabaci* at different feeding exposure

| **Feeding exposure** | **Ingestion** | | **Egestion** | |
| --- | --- | --- | --- | --- |
|  | **ToLCNDV** | **ChiLCV** | **ToLCNDV** | **ChiLCV** |
| **1 min** | 0.00E+00 | 0.00E+00 | 0.00E+00 | 0.00E+00 |
| **5 min** | 4.10E+09 (±5.29E+08) | 2.10E+11 (±1.47E+11) | 2.20E+10 (±5.29E+08) | 2.10E+09 (±1.47E+11) |
| **10 min** | 6.53E+09 (±1.87E+09) | 8.37E+11 (±5.91E+11) | 3.52E+10 (±1.87E+09) | 3.22E+09 (±5.91E+11) |
| **20 min** | 3.38E+10 (±1.08E+10) | 2.77E+12 (±1.95E+12) | 7.69E+10 (±1.08E+10) | 2.92E+10 (±1.95E+12) |
| **1 h** | 4.92E+10 (±2.32E+10) | 9.16E+12 (±6.47E+12) | 3.67E+11 (±2.32E+10) | 1.09E+11 (±6.47E+12) |
| **2 h** | 3.25E+11 (±7.6E+10) | 2.85E+13 (±2.01E+13) | 5.52E+12 (±7.6E+10) | 6.02E+11 (±2.01E+13) |
| **6 h** | 2.05E+12 (±6.1E+11) | 7.86E+13 (±5.45E+13) | 4.19E+13 (±6.1E+11) | 3.38E+12 (±5.45E+13) |
| **12 h** | 3.33E+13 (±1.31E+13) | 4.09E+15 (±2.88E+15) | 1.05E+15 (±1.31E+13) | 2.10E+13 (±2.88E+15) |
| **24 h** | 4.64E+14 (±2.54E+14) | 6.40E+15 (±4.19E+15) | 9.80E+15 (±2.54E+14) | 8.31E+13 (±4.19E+15) |

Note: The means are of three biological replicates and three technical replicates. The value in parenthesis is standard error of mean.
