# Supplementary material for: How many begomovirus copies are acquired and inoculated by its vector, whitefly (Bemisia tabaci) during feeding?
Source: PLoS One. 2021 Oct 26;16(10):e0258933. doi: 10.1371/journal.pone.0258933 (PMC8547624; doi:10.1371/journal.pone.0258933)
Supplement: S1 Fig — Melt curves of ToLCNDV (a) and ChiLCV (b) amplicons in real-time PCR analysis indicated specificity of the reactions. The specific melting temperature for both ToLCNDV and ChiLCV products was around 81°C. Standard curves show a linear relationship between log DNA concentrations in ng on X-axis and CT values on Y-axis for ToLCNDV (c) and ChiLCV (d). Each concentration was replicated thrice. The equation of the straight line and the coefficient of correlation (R2) are mentioned on the graph. (DOCX) [file pone.0258933.s002.docx]

**(b)**

**(a)**


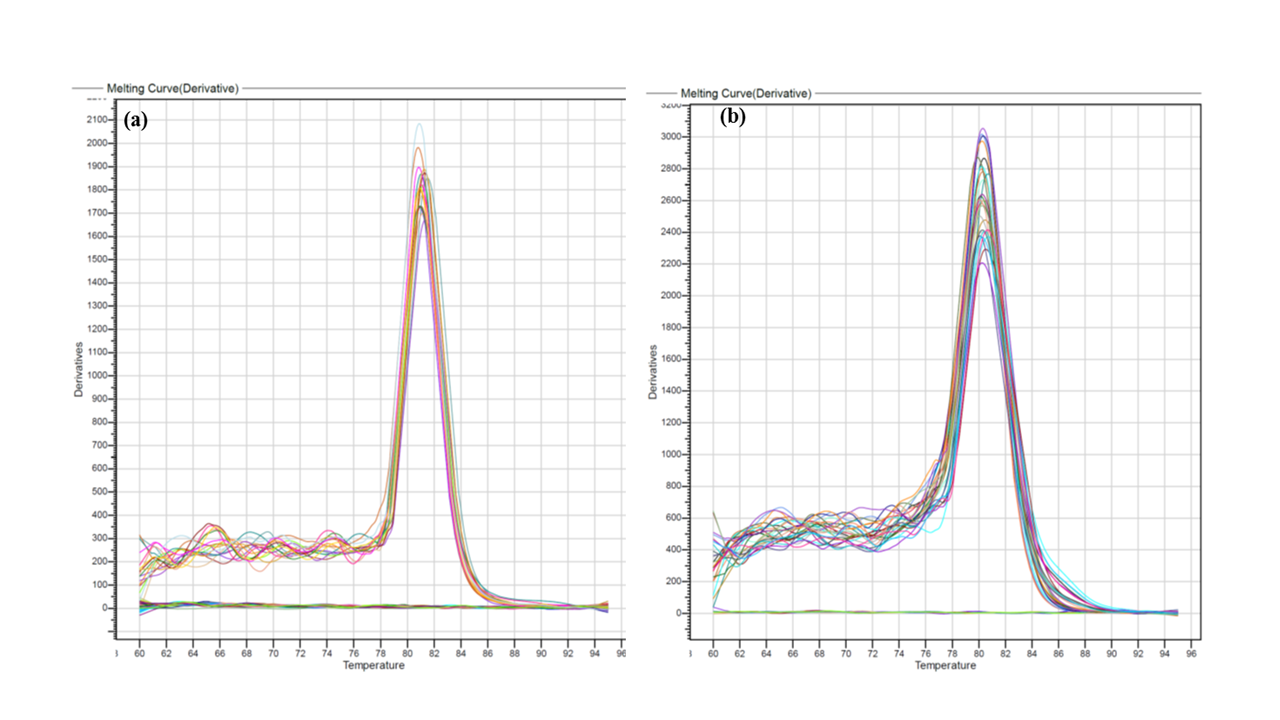


**(d)**

**(c)**

**Supplementary Fig. 1** Melt curves of ToLCNDV (a) and ChiLCV (b) amplicons in real-time PCR analysis indicated specificity of the reactions. The specific melting temperature for both ToLCNDV and ChiLCV products was around 81 °C. Standard curves show a linear relationship between log DNA concentrations in ng on X-axis and C_T_ values on Y-axis for ToLCNDV (c) and ChiLCV (d). Each concentration was replicated thrice. The equation of the straight line and the coefﬁcient of correlation (R^2^) are mentioned on the graph.
