# Supplementary material for: How many begomovirus copies are acquired and inoculated by its vector, whitefly (Bemisia tabaci) during feeding?
Source: PLoS One. 2021 Oct 26;16(10):e0258933. doi: 10.1371/journal.pone.0258933 (PMC8547624; doi:10.1371/journal.pone.0258933)
Supplement: Attachment — Submitted filename: review.pdf [file pone.0258933.s003.pdf]

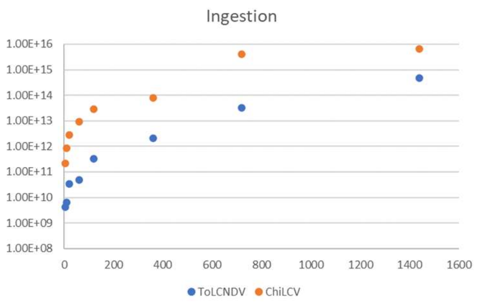
Ingestion
1.00E+16
1.00E+15
1.00E+14
1.00E+13
1.00E+12
1.00E+11
1.00E+10
1.00E+09
1.00E+08
0
200
400
600
800
1000
1200
1400
1600
ToLCNDV
ChiLCV

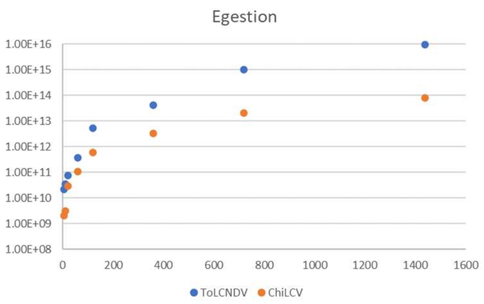
Egestion
1.00E+16
1.00E+15
1.00E+14
1.00E+13
1.00E+12
1.00E+11
1.00E+10
1.00E+09
1.00E+08
0
200
400
600
800
1000
1200
1400
1600
ToLCNDV
ChiLCV
