# Supplementary material for: How many begomovirus copies are acquired and inoculated by its vector, whitefly (Bemisia tabaci) during feeding?
Source: PLoS One. 2021 Oct 26;16(10):e0258933. doi: 10.1371/journal.pone.0258933 (PMC8547624; doi:10.1371/journal.pone.0258933)
Supplement: Attachment — Submitted filename: Response to reviewers.docx [file pone.0258933.s005.docx]

**Response to reviewers’ comments:**

We are thankful to the Reviewers and Academic Editor for critically going through the MS and suggesting substantial improvements. All the suggestions made by the reviewers have been incorporated into the revised MS. Our response to each comment is shown below. The changes made in the manuscript are indicated in red colour in the text. We trust that our revisions will make this MS acceptable for publication in the journal PLOS One.

**Reviewer #1:** Roy et al. describes in this manuscript the quantification of the ingestion and egestion by Bemisia tabaci of two begomovirus species. The authors first identify the specific biotype of B. tabaci by PCR and sequence analysis of the mtCOI gene. They then design a experimental setup that allowed them to sample B. tabaci individuals that acquire begomoviruses, Tomato leaf curl New Delhi virus or Chilli leaf curl virus, by feeding on tomato and chili leaves, respectively. Virus egestion over time is also determined. Both viruses are quantified by qPCR in individual insects.
The authors determine that the dynamics of ingestion and egestion of the two viruses are differential. In the case of ToLCNDV, the dynamic curve of ToLCNDV ingestion follows a 3er order polynomial curve, which could indicate a saturation of the virus binding sites in the insect feeding apparatus. In the egestion of this virus and in the ingestion and egestion of ChLV, the dynamics obey a 2nd order polynomial equation.

The novelty of this study lies in the quantification of the viruses in individual insects and the identification of distinct dynamics of the ingestion and release of two viruses by B. tabaci.

In my opinion, these are interesting results that deserve publication, but first it is necessary to correct some aspects of the presentation and interpretation of the results.

**Minor comments**
**Comment 1:** Figures 1 and 2 and Table 1 may go better in Supplementary material. Table 2 is graphically represented in Figure 3, so it is redundant, and is therefore either deleted or included in the Supplementary material.

**Response**: Figure 1 and 2 has been merged with Fig 3 as a single figure. Table 2 is graphically represented in Figure 3, so it has been revised as Supplementary material.

Table 1 contains primer sequences that have been designed and validated in this study. We prefer to keep this along with the main ms.

**Major comments**
**Comment 2:** In Figure 3 the curves are not well distinguishable due to the type of representation. For a better visualization, the axis of the virus titers should be in logarithmic scale. I attach some figures of how they should be. Data points should be not connected with lines and should be plotted including the standard deviations of the means. On the other hand, any number of points can be connected to a polynomial equation, but this does not help its mechanistic interpretation. Since ingestion involves adsorption to a surface/protein (virion binding sites), it should be analyzed according to adsorption models such as the Langmuir, Freundlich or Langmuir-Freundlich isotherm models, just to cite some of the most common (e.g. see: 10.1016/j.jconhyd.2011.12.001). Once the type of isotherm that best fits the data (higher R2 value) has been obtained, the curve would be included in the same figure where the real data are represented. In this way, the ingestions (binding) and releases of both viruses could be interpreted mechanistically.

**Response**: We agreed with the reviewer’s suggestion and have gone through the suggested literatures. However, the Langmuir, Freundlich or Langmuir-Freundlich isotherm models are adoptable for quantity of a gas adsorbed into a solid surface. In our case, we have virus quantity as one factor and another factor is time. Although the virus quantity adsorbed on the surface of the receptor, receptor surface area is not known in our study. Hence, the model could not be fitted in our data set. The time points of the present study could not be transformed in the model parameter.

**Reviewer #2:** It is a good study to determine the number of virions acquired and transmitted by B.tabaci to infect plants with the two begomoviruses used in the study.

The MS needs minor revision to address some questions raised in the attached copy of the MS

**Comment**: Introduction needs to be rewritten to give more information about the transmission of Begomoviruses by whiteflies.

**Response**. Necessary information has been added as suggested.

**Comment**: Please check and correct, Criniviruses, Ipomoviruses, and torradoviruses are shown to be transmitted by *B. tabaci*.

**Response**: included in the revised ms.

**Comment**: These references are not good here!!!

**Response**: Appropriate references have been cited in the revised ms.

**Comment**: Check and use the correct number

**Response**: revised as per latest ICTV checklist

**Comment**: Number not kinetics? Perhaps you want to say “The number of begomovirus particles acquired and transmitted by individual B. tabaci is important to shed light on the efficiency of virus transmission and useful in understanding the disease epidemiology.” Please check an correct.

**Response**: Revised as suggested

**Comment**: You have used similar conditions for the acquisition and inoculation of the viruses. Therefore the two paragraphs can be combined easily mentioning that for aquisition infected leaves for used and for inoculation healthy leaves were used

**Response**: merged as suggested

**Comment**: How was the uniformity in size and weight of the leaves was maintained?

**Response**: Leaves of uniform shape and size were considered throughout the experiment. Mentioned in revised ms.

**Comment**: Why not give actual calculated number instead of the formula as in the abstract you are talking about a million copies!!!

**Response**: This is not a formula. Because superscripted exponents like 10^7^ cannot always be conveniently displayed, the letter E (or e) is often used to represent "times ten raised to the power of" (which would be written as "× 10^n^").

**Comment**: What was the difference in the number of copies in tomato and chili plants used for acquisition?

**Response**: The virus copies in source plants were almost equal. Now mentioned in revised ms.

**Comment**: This needs explanation!!!

**Response**: included in discussion (line 282-291)

**Comment**: Did it match with the virus found in the whiteflies at different acquisition times?

**Response**: Virus copies increased with exposure in both acquisition and inoculation. However, the trend in increase was significantly different. Now, mentioned in discussion of revised ms.

**Comment**: How did you compensate the multiplication of the viruses in the inoculated leaves?

**Response**: the multiplication of the virus in detached leaves cannot be compensate in the current methods. This has been mentioned in discussion (line 300-310).

**Comment**: Did you test the leaves for virus titre after one minute inoculation feed at different time intervals to determine the minimum number of particles required for causing infection

**Response**: We agree with the reviewer’s suggestion. This would be an interesting study to understand the number of particles required to cause infection. However, this was beyond the scope of the current study. We will try to address this suggestion in our future studies.

**Comment**: Change to ‘vectors’

**Response**: changed

**Comment**: Change to a large number of viruses. There is no sanctity of the number 100!!! Even tomato is affected by nearly 100 species of begomoviruses.

**Response**: Changed

**Comment**: Authors should check the whole MS and be consistent. Compare this with the statement in the introduction.

**Response**: revised accordingly throughout the ms

**Comment**: You may use virions at other places too, but make it uniform.

**Response**: mentioned as virions in all places as suggested

**Comment:** Needs checking in the light of comments given earlier

**Response**: revised accordingly

Comment: Do you mean to say a million copies are required to cause infection?Please check and give convincing evidence.

**Response**: Revised as suggested

**Comment**: This is important, you need to check and confirm.

**Response**: Yes, we agree with the reviewers. Confirming the phloem contact time needs further in detail experiments and specialized instruments that is not feasible at this moment.

**Comment**: This may vary from virus-host combinations. Just quoting one reference may not be correct. Moreover, this is not relevant here.

**Response**: deleted in revised version

**Comment**: Interesting this should have prompted testing at two minutes!!!

**Response**: This may vary with host-virus-vector combinations. In the present experiment, we have tested at 1 min and 5 min.

**Comment**: Needs checking; see earlier comment. Please give actual calculated number

**Response**: It is the actual number. ‘E+10’ means 10^10^

**Comment**: Needs checking?

**Response**: The polynomial model has been removed as suggested by reviewer 1

**Comment**: Is it first time? What about WmCSV?

**Response**: The copy number of WmCSV was estimated in group of whitefly. The present study is the first to estimate the virus copies in individual whitefly

**Comment**: How?

**Response**: expanded in revised ms.

**Reviewer #3:**

It is a good piece of work which provided the information about the copy number acquired and egested by a single whitefly. Well laid out experiments brought out this authentic information and this findings support the earlier theory that a single whitefly can efficiently transmit the virus to a susceptible host which in turn can be a source of inoculam for fast spreading in the field.
Why there is a sudden fluctuation in copy number in case of ToLCNDV between 10 min to 6h of aquisition as well as in case of ChiLCV during 10 min to 12 hrs of aquisition? whether virus propagation taken place with in the body and copy number increase occurred or not during the time point between aquistion and egestion. If information available on this may be added in the manuscript

**Response**: This is not a sudden increase if you consider the duration of the virus exposure. The copies increased in individual whitefly with increase in acquisition time. The exposure to virus source for 6 to 12 hrs is the reason for increase in virus titre.

Replication of TYLCV in whitefly has been reported. We have tested the same phenomenon for predominant begomovirus species in India in a different study. There was no evidence of replication of ToLCNDV and ChiLCV in whitefly.
