# Supplementary material for: How many begomovirus copies are acquired and inoculated by its vector, whitefly (Bemisia tabaci) during feeding?
Source: PLoS One. 2021 Oct 26;16(10):e0258933. doi: 10.1371/journal.pone.0258933 (PMC8547624; doi:10.1371/journal.pone.0258933)
Supplement: Attachment — Submitted filename: Review comments-09.22.21.docx [file pone.0258933.s006.docx]

This study from Roy et al describes a novel method for quantification of acquisition & inoculation ToLCNDV and ChiLCV by Bemisia tabaci through detached leaf assay using real-time PCR. Overall, the experiments are well conceptualized and are of good scientific interest for future research on virus-vector epidemiology. The revised version of submitted manuscript has included major comments addressed from the three reviewers therefore, I recommend accepting the manuscript for publication. However, there are minor suggestions which should be incorporated in the final version.

1- The primers in Table-1 should be cross-checked as the sequence for AG153F and AG154R cannot be exact same therefore should be corrected. Also, the AG151F and AG153F are also similar, therefore please cross-confirm and apply corrections.

2- I would highly recommend the authors to provide a flow diagram or pictoral schematic template drawing for the entire protocol of ingestion and egestion by B. tabaci using detached leaf assay for better demonstration in the manuscript.

3-Please include error bars wherever applicable in the graphical data plots.
