# Supplementary material for: How many begomovirus copies are acquired and inoculated by its vector, whitefly (Bemisia tabaci) during feeding?
Source: PLoS One. 2021 Oct 26;16(10):e0258933. doi: 10.1371/journal.pone.0258933 (PMC8547624; doi:10.1371/journal.pone.0258933)
Supplement: Attachment — Submitted filename: response to reviewers.docx [file pone.0258933.s007.docx]

The authors are thankful to the Editor and reviewers for critically going htrough the manuscript and suggesting substantial improvement. All the suggestions made by the reviewers have been incorporated in the revised manuscript (R2). Our response to each comment is shown below. The changes made in the manuscript are indicated in red in the text. We trust that our revisions will make this MS acceptable for publication in Plos One.

**Reviewer #1:** Figure 1 has not been modified as suggested: Mean virus copies in the Y-axis should be in logarithmic scale for better visualization. Besides, figure 1c, 1e and 1f should be apart as e.g. figure 2, as they deal with different matters to those in figs. 1a and 1b. Nevertheless, I better think that Figs. 1c, 1e and 1f could be in Supplementary material.

**Response**: Mean virus copies have been transformed in log scale as suggested. Fig. 1c-f have been revised as supplementary materials.

To understand mechanistically the binding of viral particles and their egestion, the adsorption models outlined in my previous comments might still be useful, see Powell et al., 2000. Investigating the Effect of Carbon Shape on Virus Adsorption. https://doi.org/10.1021/es991097w. However, it is true that it is difficult to estimate the binding surfaces on the insect feeding apparatus, which might be too speculative. Therefore, I agree with the authors that their models of acquisition and egestion with time are good enough.

**Response**: Thank you. In accordance with the previous comment, the model has also been changed to a logarithmic model as the polynomial model did not fit in the revised figure after transformation of mean copy number into log scale.

**Reviewer #4:** This study from Roy et al describes a novel method for quantification of acquisition & inoculation ToLCNDV and ChiLCV by Bemisia tabaci through detached leaf assay using real-time PCR. Overall, the experiments are well conceptualized and are of good scientific interest for future research on virus-vector epidemiology. The revised version of submitted manuscript has included major comments addressed from the three reviewers therefore, I recommend accepting the manuscript for publication. However, there are minor suggestions which should be incorporated in the final version.

1- The primers in Table-1 should be cross-checked as the sequence for AG153F and AG154R cannot be exact same therefore should be corrected. Also, the AG151F and AG153F are also similar, therefore please cross-confirm and apply corrections.

**Response**: Corrected in the revised ms.

2- I would highly recommend the authors provide a flow diagram or pictoral schematic template drawing for the entire protocol of ingestion and egestion by B. tabaci using detached leaf assay for better demonstration in the manuscript.

**Response**: A pictorial diagram (Fig 1) has been provided as suggested.

3-Please include error bars wherever applicable in the graphical data plots.

**Response**: The figure has been revised in light of the comments made by Reviewer 1. SEm values are included in Supplementary Table 1.
